# Neural signatures of data-driven psychopathology dimensions at the transition to adolescence

**DOI:** 10.1192/j.eurpsy.2021.2262

**Published:** 2022-01-24

**Authors:** Amirhossein Modabbernia, Giorgia Michelini, Abraham Reichenberg, Roman Kotov, Deanna Barch, Sophia Frangou

**Affiliations:** 1 Department of Psychiatry, Icahn School of Medicine at Mount Sinai, New York, New York, USA; 2 Department of Biological & Experimental Psychology, Queen Mary University of London, London, UK; 3 Semel Institute for Neuroscience and Human Behavior, University of California Los Angeles, Los Angeles, CA, USA; 4 Department of Environmental Medicine and Public Health, Icahn School of Medicine at Mount Sinai, New York, New York, USA; 5 Seaver Center for Autism Research and Treatment, Icahn School of Medicine at Mount Sinai, New York, New York, USA; 6 Mindich Child Health and Development Institute, Icahn School of Medicine at Mount Sinai, New York, New York, USA; 7 Department of Psychiatry and Behavioral Health, Stony Brook University, Stony Brook, New York, USA; 8 Department of Psychological and Brain Sciences, Washington University in St. Louis, St. Louis, Missouri, USA; 9 Department of Psychiatry, Washington University School of Medicine, St. Louis, Missouri, USA; 10 Department of Radiology, Mallinckrodt Institute of Radiology, Washington University School of Medicine, St. Louis, Missouri, USA; 11 Djavad Mowafaghian Centre for Brain Health, Department of Psychiatry, University of British Columbia, Vancouver, British Columbia, Canada

**Keywords:** Adolescence, development, neuroimaging, population neuroscience, psychopathology

## Abstract

**Background:**

One of the challenges in human neuroscience is to uncover associations between brain organization and psychopathology in order to better understand the biological underpinnings of mental disorders. Here, we aimed to characterize the neural correlates of psychopathology dimensions obtained using two conceptually different data-driven approaches.

**Methods:**

Dimensions of psychopathology that were either maximally dissociable or correlated were respectively extracted by independent component analysis (ICA) and exploratory factor analysis (EFA) applied to the Childhood Behavior Checklist items from 9- to 10-year-olds (*n* = 9983; 47.8% female, 50.8% white) participating in the Adolescent Brain Cognitive Development study. The patterns of brain morphometry, white matter integrity and resting-state connectivity associated with each dimension were identified using kernel-based regularized least squares and compared between dimensions using Spearman’s correlation coefficient.

**Results:**

ICA identified three psychopathology dimensions, representing opposition–disinhibition, cognitive dyscontrol, and negative affect, with distinct brain correlates. Opposition–disinhibition was negatively associated with cortical surface area, cognitive dyscontrol was negatively associated with anatomical and functional dysconnectivity while negative affect did not show discernable associations with any neuroimaging measure. EFA identified three dimensions representing broad externalizing, neurodevelopmental, and broad Internalizing problems with partially overlapping brain correlates. All EFA-derived dimensions were negatively associated with cortical surface area, whereas measures of functional and structural connectivity were associated only with the neurodevelopmental dimension.

**Conclusions:**

This study highlights the importance of cortical surface area and global connectivity for psychopathology in preadolescents and provides evidence for dissociable psychopathology dimensions with distinct brain correlates.

## Introduction

One of the challenges in human neuroscience is to uncover associations between brain organization and psychopathology in order to better understand the biological underpinnings of mental disorders. As individuals typically present with a range of behavioral or self-reported problems, statistical modeling can be used to extract latent dimensions of psychopathology [1]. The assumptions of these statistical models vary which could be consequential for uncovering biologically meaningful phenomenon. A widely used approach involves the application of factor analytic models to identify dimensions (termed factors) allows for uncovering correlated constructs [2]. The dimensions specified by the Hierarchical Taxonomy of Psychopathology (HiTOP) model is a key example [3,4]. Conversely, application of independent component analysis (ICA) decomposes the variance of individual ratings to yield maximally dissociable dimensions (termed components) [5–10]. In this context, ICA can uncover statistically independent psychopathology constructs while still allowing for individual symptoms to be shared across dimensions. These dimensions can then be leveraged to identify potentially distinct biological mechanisms underlying psychopathology. As an example, independent dimensions of psychopathology in youth participating in the Philadelphia Neurodevelopmental Cohort have been associated with distinct patterns of white matter connectivity [8].

Late childhood and adolescence is a period of particular interest in the contest of brain-psychopathology associations because it coincides with major brain maturational changes [11–14] and with the epidemiological peak for incident mental disorders [15,16]. Accordingly, we focus on brain-psychopathology associations in a population sample of 9–10-year-olds participating in the multisite USA study of Adolescent Brain Cognitive Development (ABCD) [17,18]. The ABCD study has a longitudinal design aiming to capture changes in brain, behavior and cognition as participants traverse through adolescence. Here, we use baseline data to determine brain-psychopathology associations at the point of transition to adolescence. Participants’ psychopathology was rated using the items of the Child Behavior Checklist (CBCL) [19] to which we applied both factor and independent components analyses with the aim of discovering brain features associated with the dimensions identified by the two different models. The working hypothesis was that decomposition of psychopathology into independent components, as opposed to factors, would offer more mechanistic insights by identifying brain properties relating to morphometry and connectivity that are differentially associated with psychopathology.

## Methods

### Sample

The ABCD baseline sample comprises 11,875 individuals aged 9–10 years enrolled at 22 sites (https://abcdstudy.org/) (Supplemental Methods, Section 1). The analyses presented here used data from 9983 unrelated participants with an average age of 9.9 years (47.8% female, 50.8% white). The ABCD study was approved by the institutional review board of the University of California, San Diego.

### Measures

#### Psychopathology

Parents rated their children’s behavior over the preceding 6-months based on the 119 items of the CBCL (Supplemental Methods, Section 2.1, Supplementary Table S1).

#### Child characteristics

We used a wide range of measures pertaining to cognition, academic functioning psychological traits, and mental health service utilization (Supplemental Methods, Section 2.2, Supplementary Table S2) for the external validation of the psychopathology dimensions.

#### Neuroimaging measures

ABCD participants underwent structural magnetic resonance imaging (sMRI), diffusion MRI (dMRI), and resting-state functional MRI (rs-fMRI) using standardized acquisition and analyses protocols (Supplemental Methods, Section 3). We downloaded preprocessed data from the ABCD repository to analyze features of morphometry (cortical thickness, surface area, and subcortical volume), white matter integrity (fractional anisotropy and mean, radial, and axial diffusivity), and cortical resting-state networks (RSN) (detailed description of the neuroimaging variables in Supplementary Tables S3–S5).

### Statistical Analysis

#### Extraction of psychopathology dimensions

All dimensions of psychopathology were extracted from the CBCL items using either ICA or exploratory factor analyses (EFA). The EFA-derived factors were derived by Michelini et al. for a prior study on the ABCD dataset [20]. Both ICA and EFA are exploratory methods that can represent data hierarchically. The difference between the two methods is that ICA assumes statistical independence of the extracted dimensions, whereas EFA if used with an oblique rotation allows for a correlated factor structure.

ICA was implemented using the Big Omics Data computational pipeline (https://github.com/LabBandSB/BIODICA) [5,21]. Models with 2–10 components were generated and the optimal solution was chosen based on stability through 100 random runs. Stability across sites was established using a leave-one-site-out approach (details provided in Supplemental Methods, Section 5). The derived psychopathology dimensions were further characterized by examining their Neurobehavioral and Functional Profile. The EFA dimensions were provided by Michelini et al. [20] based on their analyses of the ABCD dataset (Supplemental Methods, Section 5). Briefly, in the EFA, the hierarchical structure of psychopathology was explored by applying principal component analysis to the matrix of polychoric correlation between CBCL items and using an oblique rotation (geomin) to extract factor solutions with an increasing number of factors. The maximum number of factors were determined using parallel analysis while ensuring factor interpretability.

#### Neuroimaging correlates of psychopathology dimensions

We used a series of kernel-based regularized least squares (KRLS) analyses [22,23] to quantify the associations between each ICA- and EFA-derived psychopathology dimension and the neuroimaging variables. We chose KRLS because it does not make assumptions about the shape of the associations (i.e., linear or nonlinear) and is easily interpretable. KRLS yields average effect estimates of the independent variables, analogous to the beta coefficients of linear regression models. KRLS models were conducted separately for each modality (i.e., sMRI, dMRI, and rs-fMRI). For the sMRI and dMRI models, both global (i.e., mean cortical thickness, total surface area and total intracranial volume, and average fractional anisotropy and diffusivity) and regional measures were used as predictors; in the rs-fMRI models, measures of between- and within-network connectivity were used as predictors. All models were adjusted for sex, age, and race, handedness, scanner, weight, height, pubertal stage while motion was also included in models considering rs-fMRI and dMRI variables. The analyses were not adjusted for total intracranial volume (TIV), because we consider TIV as a marker of global neurodevelopment. However, by adjusting for demographic and anthropometric variables, we controlled for the portion of TIV that is determined by nonneurodevelopmental factors.

To establish that the results of all KRLS models were independent of sample composition all analyses were repeated 100 times, each time by randomly resampling 50% of the total dataset. Subsequently, the *t*-value vectors (regardless of significance) obtained from the KRLS models for each psychopathology dimension were compared using Spearman’s correlation coefficient. The stability of the associations was judged based on the number of times they were statistically significant at *p*_False Discovery Rate (FDR)_ ≤ 0.05. The direction of association was informed by the original model with the full dataset.

## Results

### Psychopathology dimensions

**Figure 1.**
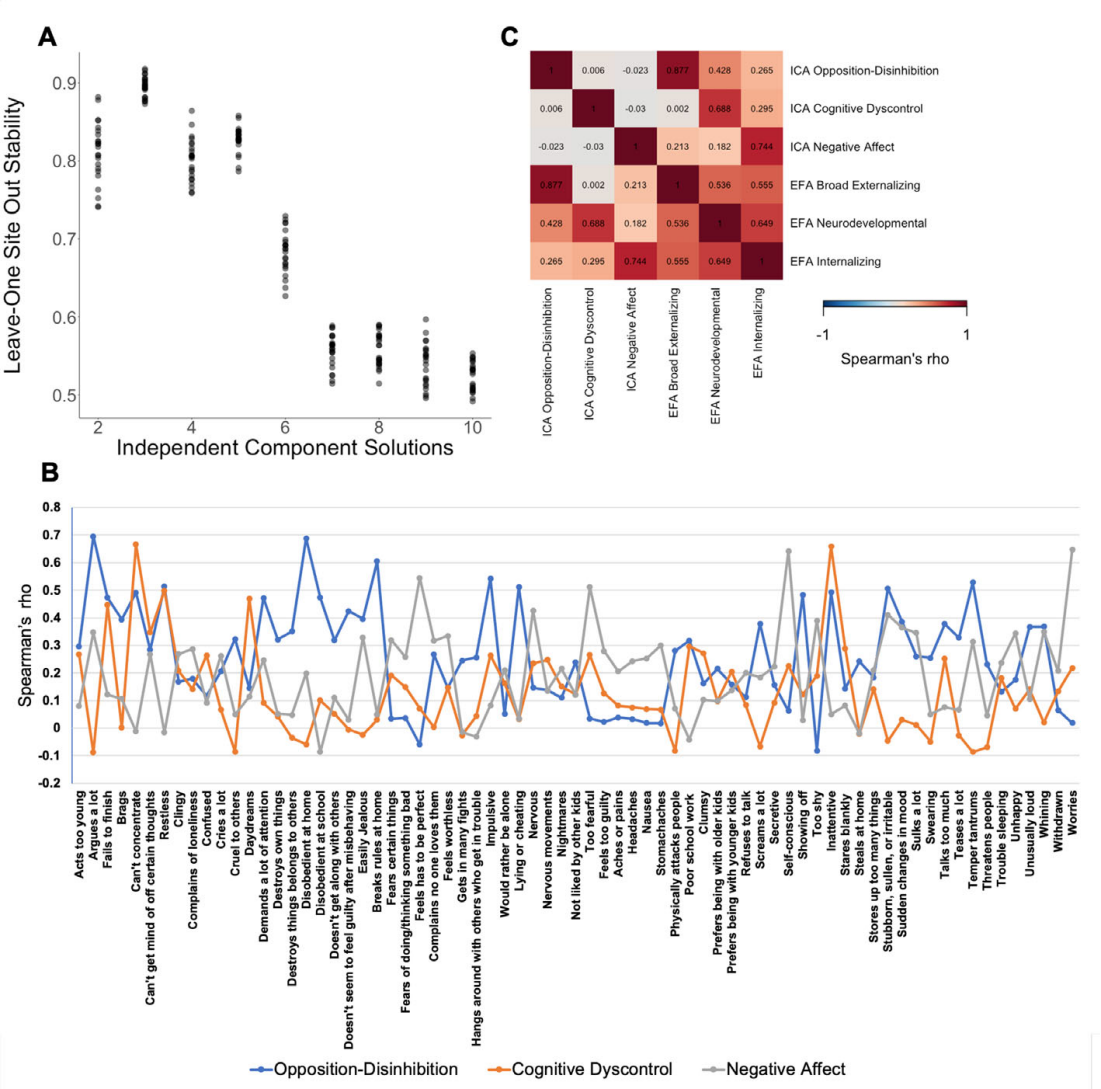
Data-driven structure of psychopathology in the ABCD dataset captured independent component analysis (ICA). A. Leave-one site stability for ICA solutions from 2-10 components; B. Item loadings on each independent component for the three-IC solution; C. Correlation between the three-IC solution and corresponding EFA-derived factors.

**Figure 2.**
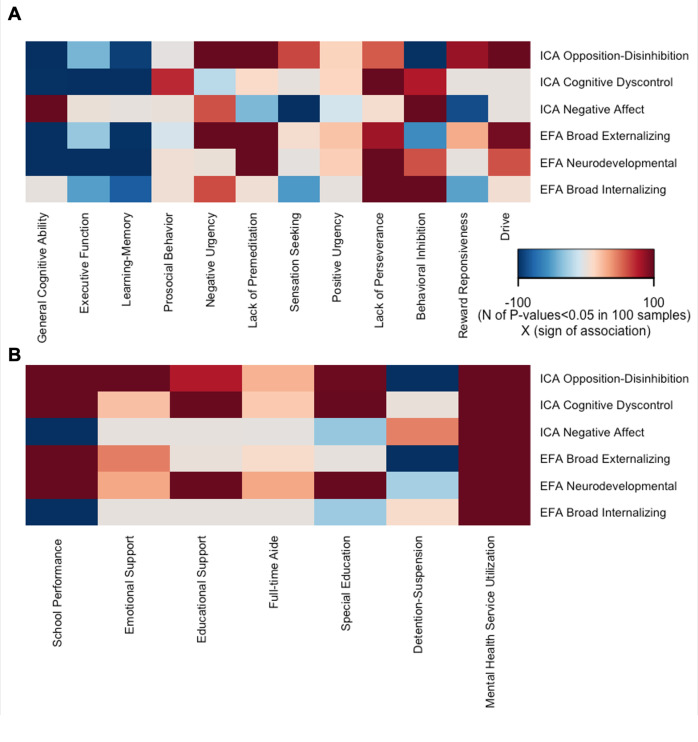
Validity of the Psychopathology Dimensions. Each cell represents number of FDR-corrected P-values <0.05 in 100 reruns of the Kernel regularized least squares (KRLS) analysis on 50% randomly resampled data multiplied by the sign of the association. All analyses were adjusted for sex, race, age, and site; A. Association of psychopathology dimensions with cognitive and behavioral measures; for each psychopathology dimension, all measures are modeled together to characterize their independent contribution to psychopathology; B. Association of psychopathology dimensions with academic outcomes and service utilization; for each outcome, all three dimensions (ICA-derived or EFA-derived) are entered into the model together to quantify their independent contribution to the outcome.

Amongst the plausible the ICA solutions, the three-component solution was maximally stable (stability = 0.90, leave-one-site-out stability of 0.85–0.92, Figure 1A) and minimally correlated (Spearman’s *ρ* = −0.03 to 0, Figure 1B) (Supplemental Results, Section 1; Supplementary Figures S1–S3). Based on the items with the highest loadings, these three dimensions represented opposition–disinhibition, cognitive dyscontrol, and negative affect (Supplementary Table S7). In the opposition–disinhibition dimension, CBCL items relating to rule breaking, impulsivity, and hostility-aggression had the highest loadings. In the cognitive dyscontrol dimension, high-loading CBCL items related to inattention, poor concentration, and restlessness, and in the negative affect dimension, high-loading CBCL items reflected fears, sadness, worries, and social discomfort. The opposition–disinhibition dimension was mainly associated with poorer general cognitive ability, whereas cognitive dyscontrol was associated with lower performance across cognitive domains; both opposition–disinhibition and cognitive dyscontrol were associated with poorer academic function (Figure 2, Supplemental Results, Section 2; Supplementary Tables S8 and S9 and Supplementary Figure S4) but the opposite was the case for the negative affect dimension.

To allow direct comparison with the three-component ICA solution, the EFA dimensions considered were the 3-factor solution consisting of broad externalizing dimension, the neurodevelopmental problems dimension, and broad internalizing dimension which showed marked correlation (Spearman’s *ρ* = 0.53–0.65). The highest loading CBCL items on the broad externalizing, the neurodevelopmental, and broad internalizing dimensions were similar to the opposition–disinhibition, cognitive dyscontrol, and negative affect dimensions, respectively (Figure 1C and Supplementary Tables S8 and S9). A comparison of the most fine-grained solutions for both ICA and EFA (consisting of five dimensions each) is provided in Supplementary Figures S20–S29.

The broad externalizing and neurodevelopment dimensions were associated with lower cognitive tasks performance and academic function while the broad internalizing dimension was positively associated with behavioral inhibition and lack of perseverance (Figure 2, further details in Supplementary Tables S8 and S9).

### Neuroimaging correlates of dimensional psychopathology

#### Brain morphometry

**Figure 3.**
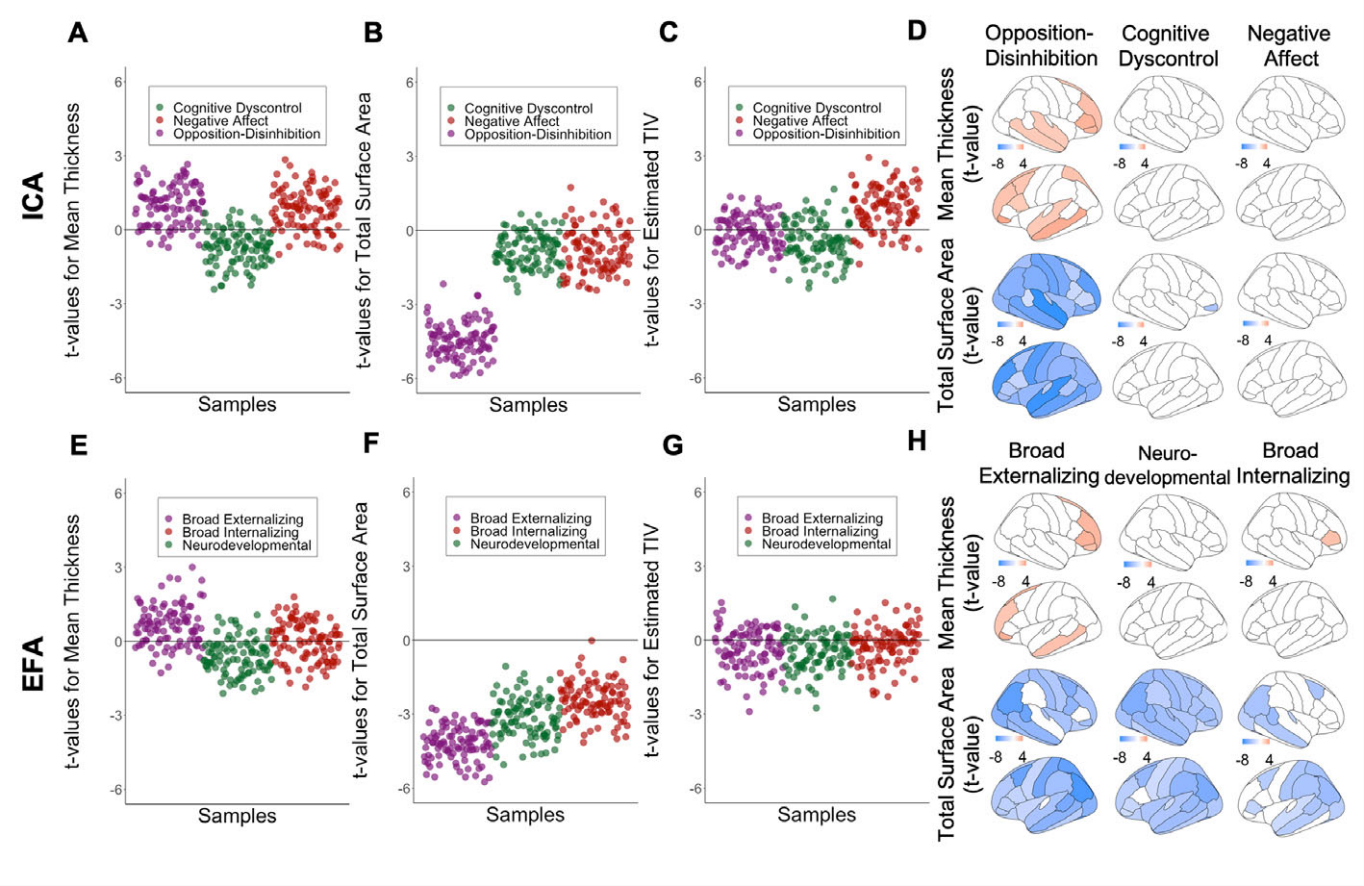
Association between global measures of brain morphometry and the most stable ICA solution, and corresponding EFA factors using kernel regularized least squares (KRLS); Values represent t-value for the association between morphometric brain measures and dimensions of psychopathology. For global measures, values represent t-values in 100 randomly resampled dataset of 50% original sample size. For regional measures values represent t-value from the model on the whole dataset; All analyses were adjusted for sex, age, and race, handedness, scanner, weight, height, pubertal stage; A, B, C Association between brain morphometric measures and ICA-derived opposition-disinhibition, cognitive dyscontrol, and negative affect dimensions. D, E, F. Association between brain morphometric measures and EFA-derived broad externalizing, neurodevelopmental, and broad internalizing factors.

With respect to the ICA-derived dimensions, opposition–disinhibition was the only dimension associated with brain morphometry involving negative associations with global and regional cortical surface area and regional subcortical volumes and a positive association with cortical thickness in frontotemporal regions (Figure 3, Supplementary Table S10, and Supplementary Figures S5, S6, and S8). By contrast, all three EFA-derived dimensions were significantly associated with the reduced surface area; the broad externalizing dimension was additionally associated with the frontotemporal thickness (Figure 3 and Supplementary Figures S5, S7, and S9). The similarity of the regional morphometric profiles between ICA-derived dimensions was low (Spearman’s *ρ* = −0.10 to 0.23) (Supplementary Figure S11) while it was very high for the EFA-derived dimensions (Spearman’s *ρ* = 0.87–0.91).

#### White matter integrity

**Figure 4.**
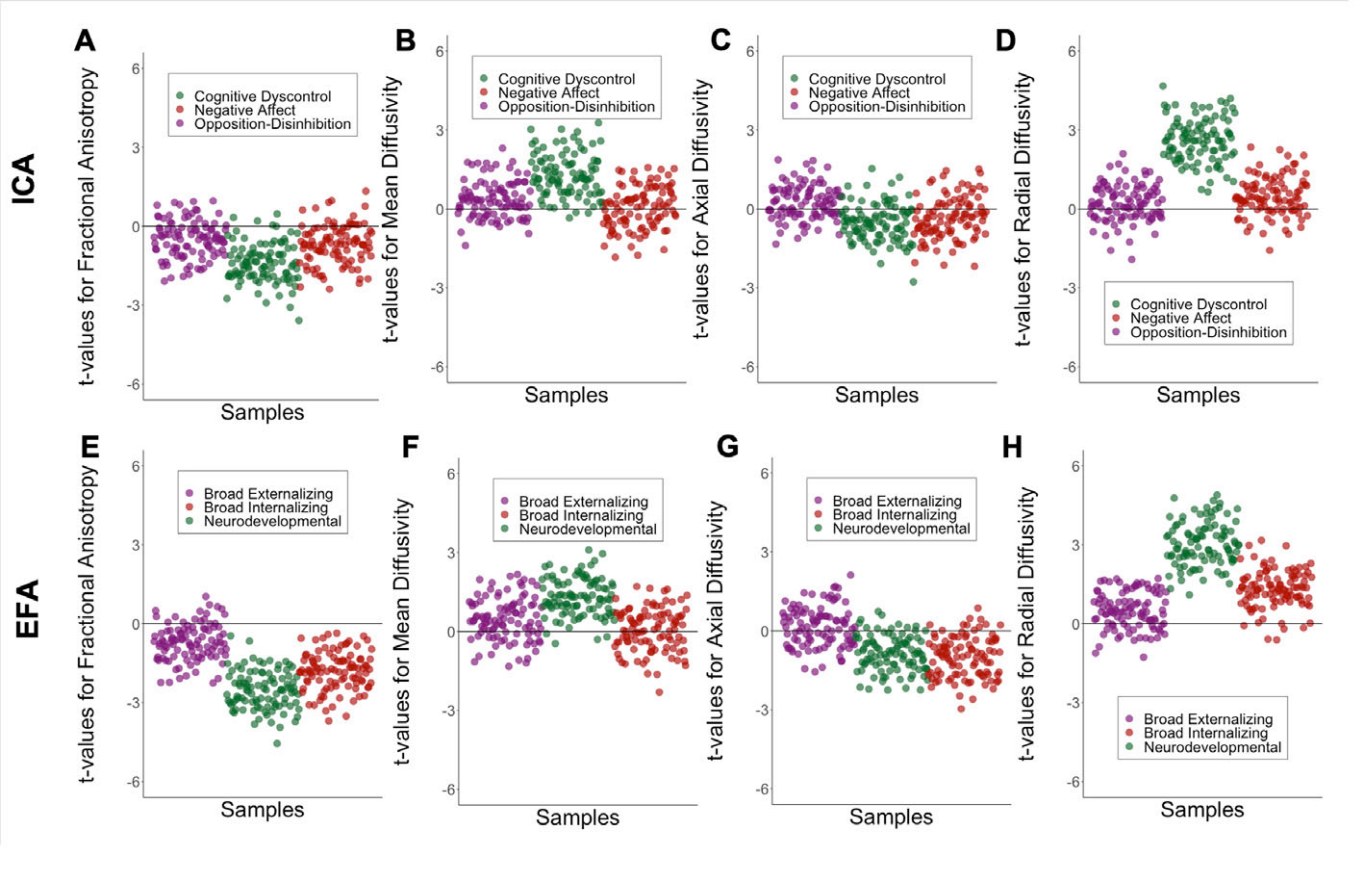
Association between global measures of white matter integrity and the most stable ICA solution, and three corresponding EFA factors using kernel regularized least squares (KRLS). Values represent t-value for the association between the measures of white matter integrity and psychopathology dimensions in 100 randomly resampled dataset of 50% original sample size; All analyses were adjusted for sex, age, and race, handedness, scanner, weight, height, pubertal stage, and head motion; A,B,C,D. Association between measures of white matter integrity and ICA-derived opposition-disinhibition, cognitive dyscontrol, and negative affect dimensions. E, F, G, H. Association between measures of white matter integrity and EFA-derived broad externalizing, neurodevelopmental, and broad internalizing factors.

The ICA-derived dimension of cognitive dyscontrol as well as the EFA-derived neurodevelopmental dimension were both associated with mean radial diffusivity and with multiple regional measures (Figure 4, Supplementary Table S11, and Supplementary Figures S12–S15). Except for negative affect and broad externalizing dimensions, all other ICA- and EFA-derived dimensions showed localized associations with dMRI measures in the superior corticostriate and corticospinal tracts (Supplementary Figures S12–S14). The pair-wise similarity in regional white matter integrity profiles was moderate for ICA-derived dimensions (Supplementary Figure S16) (Spearman’s *ρ* = 0.44–0.59) and high for EFA-derived dimensions (Spearman’s *ρ* = 0.71–0.89).

#### RSN-connectivity

Significant associations between resting-state connectivity and psychopathology were noted only for the ICA-cognitive dyscontrol and the EFA-neurodevelopmental dimensions. Cognitive dyscontrol mainly showed (a) positive associations with the internetwork connectivity of the cingulo-opercular/default mode networks, the frontoparietal/sensorimotor-hand networks, and the default mode/dorsal attention networks; and (b) negative associations with the intranetwork connectivity of the sensorimotor-hand, the default mode, and the cingulo-opercular networks (Supplementary Figures S17 and S18). Similar associations were observed between the neurodevelopmental dimension and resting state functional connectivity measures (Supplementary Figure S18). The similarity between functional connectivity profiles was low between the ICA dimensions (Supplementary Figure S19, Spearman’s *ρ* = −0.32 to 0.02) and moderate for the EFA dimensions (Spearman’s *ρ* = 0.38–0.64).

## Discussion

We leveraged the ABCD dataset with the expressed intention of delineating how different conceptualizations of psychopathology may influence associations with measures of brain organization. The most significant differences between ICA- and EFA-derived dimensions were noted for brain morphometry, where all EFA-dimensions were associated negatively with global and regional measures of cortical surface area while this was the case only for the opposition–disinhibition ICA dimension. Cognitive dyscontrol and developmental problems showed similar associations with measures of white matter integrity and functional connectivity. Negative affect and internalizing dimensions had minimal associations with neuroimaging measures.

There are three general observations arising from this study which appear to reflect general principles in brain-psychopathology associations. First, across dimensions, robust associations between psychopathology and measures of brain organization were generally spatially diffuse. Prior multivariate studies have also reported that associations between brain organization and behavior are typically global rather than regionally specific [24–26]. Second, although some brain properties showed more robust associations with specific dimensions, the overall profiles of their brain correlates showed moderate correlations; this was the case even for the ICA-derived dimensions that were statistically dissociable. Similar findings have been reported in a population sample of older individuals with respect to the genetic correlates of ICA-derived behavioral traits [7]. These findings align with the “multiple realizability” brain-behavior framework, where distinct clinical phenomena can arise as a result of neurobiological processes with considerable overlap [27]. Third, the presence of psychopathology was associated with cortical dysmaturation. Typical maturational changes involve cortical thinning and cortical surface area expansion [13,28]. Psychopathology (and primarily opposition–disinhibition and broad externalizing problems) was associated with aberrant cortical maturation indexed by negative associations between psychopathology with cortical surface area and positive associations with cortical thickness in frontal association regions involved in top-down inhibitory control. Additionally, cognitive dyscontrol was associated with lower anatomical and functional connectivity. These findings add to the current debate as to the nature of brain maturation for externalizing/conduct problems [29] and cognitive difficulties [30,31].

Arguably the most surprising finding was that the ICA-derived negative affect and EFA-derived internalizing dimensions had minimal associations with any imaging measures. Two prior studies on the ABCD sample reached the same conclusion with regards to the association between broad internalizing and RSN connectivity as well as between suicidal ideas and structural and functional brain measures [32,33]. Even amongst youths with established internalizing disorders, a significant proportion had been reported to have preserved brain structure and cognitive functioning with despite high levels of internalizing psychopathology [34]. These observations are open to several interpretations. Negative affect in youth may not be associated with significant brain abnormalities as indicated by its positive association with cognition. It could be argued that negative/internalizing problems reflect meta-cognitive alternations involving negative evaluations occurring in the context of largely intact brain organization. Alternatively, it is possible that brain properties associated with negative affect and internalizing problems are below macrolevel resolution or that detectable macrolevel changes may become apparent either at different stages of development or when they are chronic. The longitudinal design of the ABCD study will enable testing these hypotheses.

It is important to note several methodological issues. First, the ICA implementation was methodologically rigorous and included steps to optimize its stability and external validity of the dimensions identified. Second, the kernel-based approach ensures that complex association can be detected as these can be missed when linear models only are employed. Third, to address the computational costs of the analysis, the current study used atlas-based measures of brain structure and function which may involve loss of information compared to more fine-grained approach such as voxel-wise analysis. Fourth, no assumptions of causality can be made on the basis of cross-sectional data, but the study sets the foundation for future hypothesis testing in the longitudinal ABCD dataset.

In sum, detailed characterization of psychopathology undertaken in the largest available sample of preadolescents highlights cortical surface area and connectivity as the brain phenotypes with the most robust associations with psychopathology dimensions regardless of how they were defined.

## Data Availability

Data used in the preparation of this article were obtained from the Adolescent Brain Cognitive Development (ABCD) Study (https://abcdstudy.org), held in the NIMH Data Archive (NDA). The ABCD dataset is available upon request to the National Data Archive, https://nda.nih.gov/abcd.

## Supplementary Materials

To view supplementary material for this article, please visit http://doi.org/10.1192/j.eurpsy.2021.2262.
